# Modern Family Planning Utilization and Its Associated Factors among Currently Married Women in Rural Eastern Ethiopia: A Community-Based Study

**DOI:** 10.1155/2020/6096280

**Published:** 2020-12-29

**Authors:** Teshale Mulatu, Yitagesu Sintayehu, Yadeta Dessie, Merga Deressa

**Affiliations:** ^1^School of Nursing and Midwifery, College of Health and Medical Sciences, Haramaya University, P.O. Box 235, Harar, Ethiopia; ^2^Department of Midwifery, College of Medicine and Health Sciences, Dire Dawa University, P.O. Box 1362, Dire Dawa, Ethiopia; ^3^School of Public Health, College of Health and Medical Sciences, Haramaya University, P.O. Box 235, Harar, Ethiopia

## Abstract

**Background:**

The use of modern family planning methods among women of reproductive age (15-49 years) is of public health importance in Ethiopia. Nationally, modern family planning method use remains as low as 35%. Understanding factors associated with the use of modern family planning methods may help to improve maternal and child health. Hence, this study is aimed at assessing modern family planning method use and its determinants among women of reproductive age in the rural districts of Eastern Hararghe zone, Eastern Ethiopia. *Methodology*. A community-based, cross-sectional survey was conducted among 577 randomly selected, currently married, reproductive-aged women in selected rural districts of Eastern Hararghe, Eastern Ethiopia. Data were collected using a pretested, interviewer-administered questionnaire about women's sociodemographic information, knowledge about contraception, reproductive history, contraceptive use and fertility desire, couple's communication, and decision-making on family planning. Binary and multivariable logistic regression was used to analyze the association between the dependent and independent variables.

**Result:**

A total of 555 study participants participated, yielding a 96.2% response rate. The overall modern family planning utilization among the study participants was 18.4%. Knowledge of modern family planning methods (AOR = 16.958, CI: 4.768, 60.316), husband approval (AOR = 3.590, CI: 2.170, 5.936), couple's discussion (AOR = 2.852, CI: 1.759, 4.623), male involvement in decisions about family planning (AOR = 2.340, CI: 1.531, 3.576), desire for additional child (AOR = 2.295, CI: 1.528, 3.447), and previous use of contraception (AOR = 0.018, CI: 0.005, 0.063) were significantly associated with modern contraceptive utilization.

**Conclusion:**

Even though knowledge of modern family planning methods was very high, the overall modern family planning method use in the study area was low. The government should focus on increasing modern family planning method availability. It must also ensure family planning method security and create awareness on modern family planning methods through community-based education and proper counselling to empower women to make an appropriate choice.

## 1. Introduction

Family planning (FP) refers to the use of contraceptive methods to prevent unintended pregnancy, limit the number of children, and space childbirth. Contraceptive methods are classified as modern or traditional methods. Modern methods include female sterilization, male sterilization, intrauterine contraceptive device (IUD), implants, injectables, pill, male condoms, female condoms, emergency contraception, and lactational amenorrhea method (LAM), whereas traditional methods include rhythm (calendar), withdrawal, and folk methods [1].

Contraceptive has many benefits; it ensures couples achieve the desired family size and reduces infant/perinatal and maternal mortality. It also reduces the risk of HIV transmission and STI acquisition and prevents unintended pregnancies. Moreover, it decreases pregnancy and birth-related complications as it provides adequate time for a mother to recover from the previous pregnancy sufferings [2–4].

The 1994 International Conference on Population and Development (ICPD) shifted the world from one concerned with population growth to one committed agenda of reproductive rights and justice. It created a platform to help women and men to have greater access to modern contraceptive methods and affordable, convenient FP. FP is a vital element for the achievement of sustainable development goal [3], which considers reproductive, maternal, and child health as a priority agenda [5]. However, more than one in ten married or in-union women worldwide have an unmet need for family planning [3].

Currently, greater than 200 million women in developing countries want to avoid pregnancy, but they are not using any type of modern contraceptive method. Because fertility and unmet need for contraception continue to be much greater in sub-Saharan Africa , the magnitude of maternal death is much higher in this region compared to other regions. An unmet need for contraception inevitably results in unintended pregnancies. Approximately 40 percent of all pregnancies in developing countries are unintended [2, 4, 6].

Worldwide, the prevalence of modern contraceptive method use was 64% in 2015. According to the UN, contraceptive prevalence must be 66%-75% in developed countries and 67% in developing countries in order to achieve the desired fertility decline by 2025 [7]. However, this trend has not been seen in developing countries where the use of modern contraception among women of reproductive age is about 43% [8].

Modern FP utilization remains low in sub-Saharan Africa (SSA). In 2012, the contraceptive coverage and unmet need for contraception in the region were 25.7% and 25.1%, respectively [7, 9].

The 2016 Ethiopian Demographic Health Survey reported that the overall use of FP methods among currently married women was 36%: of these, 35% were using a modern method, and only 1% were using a traditional method. Data shows that 22% of currently married women have an unmet need for FP services, 13% for spacing and 9% for limiting [1].

Ethiopia has increased FP service coverage by providing various contraceptive methods at the household level. The Ethiopian government began the ongoing Health Extension Worker (HEW) program in 2003. The HEW is a community-based intervention program, which delivers disease prevention and control, family health services, hygiene and environmental sanitation, and health education and counselling [10]. FP was incorporated into the program as one of 16 essential health services provided at the community level by health extension workers. HEWs were cross-trained to work in new health outlets, a massive investment in a country as poor as Ethiopia. These auxiliary health personnel provided injectable contraception and in recent years have begun to insert implants [2].

Although Ethiopia has been mentioned as a champion for introducing HEWs who provide modern contraceptive methods at the doorstep in rural kebeles, the utilization of modern contraceptive methods remains very low [11, 12]. Research is needed to understand factors associated with the use of modern FP methods and the factors underlying modern FP utilization. Hence, the current study was conducted with the intent to assess modern contraceptive method utilization and its determinants among married women in the rural settings of the study area.

## 2. Methodology

### 2.1. Study Setting

East Hararghe is one of the zones of the Ethiopian Region of Oromia, located in the Eastern part of Ethiopia, 510 km from Addis Ababa. East Hararge is bordered on the southwest by the Shebelle River, which separates it from Bale, on the west by West Hararghe, on the north by Dire Dawa, and on the north and east by the Somali Region. The Harari Region is an enclave inside this zone. The zone has a total population of 2,723,850, of whom 1,383,198 are men and 1,340,652 are women, with an area of 17,935.40 square kilometers. East Hararge has a population density of 151.87. Only 8.27% (216,943) are urban inhabitants and 30,215 or 1.11% are pastoralists.

Approximately 580,735 households were residents of this zone at the time of the study, with an average of 4.69 persons to a household and 560,223 housing units [13]. This study was conducted on January 1-30, 2019.

### 2.2. Study Design

A community-based cross-sectional study was conducted.

### 2.3. Source and Study Population

Currently married reproductive-aged women living in rural parts of the Eastern Hararghe zone were the source population, and currently married reproductive-aged women living in selected kebeles of the study areas were the study population.

### 2.4. Sample Size Determination and Sampling Procedure

The single population proportion formula, *n* = (*Zα*/2)^2^*pq*/*d*^2^, was used with the proportion (35%) which was taken from EDHS 2016, which showed that 35% are using a modern method [1]. A confidence level of 95% and a 5% degree of precision were used. By considering the 1.5 design effect and 10% nonresponse rate, the final sample size was 577. Multistage sampling techniques were used. Among woredas (districts) in the study area, three woredas were selected by lottery methods. Then, one kebele (the smallest administrative unit) from each woredas was selected by simple random sampling. Proportional allocation for each kebele was used to give equal chance. Using systematic random sampling, all eligible households in each selected kebele were selected. From the eligible households, study participants (currently married women) were selected by simple random sampling. If there was more than one eligible woman within the selected household, one woman was picked at random. Women with mental and serious health problems, pregnant women, and those who reported infertility were excluded from the study.

### 2.5. Data Collection

Data were collected using a pretested, interviewer-administered questionnaire asking about women's sociodemographic information, knowledge about contraception, reproductive history, contraceptive use and fertility desire, couple's communication, and decision-making about FP. Three HEWs were recruited for data collection. To control the data quality, one-day training was given for supervisors and data collectors. The tools were pretested, and modifications were made to our set-up based on the pretest results. Collected data were reviewed for quality assurance on a daily basis, and double data entry control was done.

### 2.6. Data Analysis

Collected data were entered into the computer by using EpiData Version 3.0.2. Data analysis was done by using Statistical Package for the Social Sciences (SPSS) software version 21. Frequencies of variables were generated; tabulation and percentages were used to illustrate study findings. Bivariate and multivariate logistic regression analyses were used to analyze the association between the dependent and independent variables. The outcome variable (current modern FP use) was coded to yes/no response, and each explanatory variable was tested for association in bivariate analyses. Covariates with a *P* value < 0.2 were retained and entered into the multivariable logistic regression analysis using a forward selection and backward elimination approach. Hosmer and Lemeshow's goodness-of-fit test was used to assess whether the necessary assumptions were fulfilled. An adjusted odds ratio (AOR) with 95% confidence intervals (CI) using a *P* value < 0.05 was considered statistically significantly associated with the outcome variable.

### 2.7. Measures

*Modern family planning*: use of modern medicines (hormonal) or artificial material (condom) and minor surgery (voluntary sterilization) to space or limit birth.

*Current modern FP method utilization*: a woman was considered a current user if she is using any modern FP method during the survey.

*Ever use of modern FP methods*: a woman was considered ever using modern FP methods if she had used any modern FP methods previously before the survey.

*Knowledge of modern FP methods*: a woman was considered knowledgeable if she knew at least one type of modern FP method.

### 2.8. Ethical Consideration

The ethical approval was obtained from the Haramaya University College of Health and Medical Sciences institutional health research ethics review committee. Formal letters were written to all concerned authorities, and permission was secured at all levels. After explaining the purpose and procedures of the study, informed, voluntary, written, and signed consent was obtained from each respondent. All the basic principles of human research ethics (respect of persons, beneficence, voluntary participation, confidentiality, and justice) were respected.

## 3. Results

### 3.1. Sociodemographic Characteristics of Respondents

A total of 555 study participants participated in the study, with a 96.2% response rate. More than half 304 (54.8%) of the study participants were between the age group of 25-35 (mean age of 28.2 ± 6 SD). A majority 459 (82.7%) of the study participants were from the Oromo ethnic group.

Most of the study participants 471 (84.9%) were Muslim. With regard to educational status, 307 (55.3%) had no formal education, while 195 (35.1%) had attended primary school. More than one-third 242 (43.6%) had an average monthly income of <1000 ETB (Table 1).

### 3.2. Maternal and Reproductive History of Study Participants

**Figure 1:**
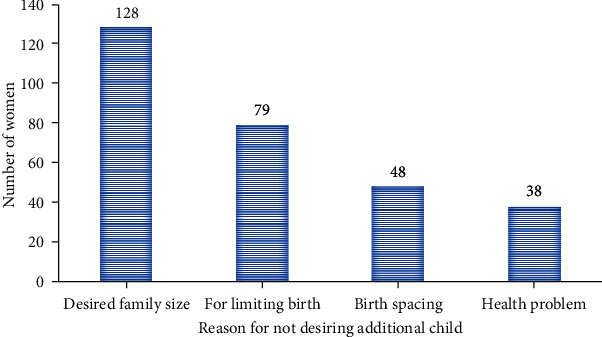
Reason for not desiring additional children among currently married women of reproductive age in selected rural areas of Eastern Hararghe zone, Eastern Ethiopia, January 1-30, 2019 (*n* = 555).

Regarding the reproductive and obstetric history of the study participants, almost all mothers 549 (98.9%) had both histories of pregnancy and childbirth. More than half of the study participants 319 (57.5%) had four or fewer children, followed by five children and above 236 (42.5%). When asked about the desire for more children, more than half of the study participants 293 (52.8%) had no desire for more children. The most cited reason for not desiring more children was having the desired number of children 128 (43.7%) followed by limiting birth 79 (27%) (Figure 1).

### 3.3. Knowledge of Modern Contraceptive Methods towards Use of Modern Contraceptive Methods

Most of the study participants 521 (93.9%) had heard about modern FP methods. The study participants heard information from health professionals 430 (80.8%) and from friends/relatives 85 (18.2%), from the church/mosque 30 (5.6%), and at school 23 (4.1%), respectively. The most popular modern contraceptive method was injection 491 (94.2%), followed by implants 447 (85.8%), pills 367 (70.4%), intrauterine contraceptive device (IUCD) 79 (15.2%), condom 24 (4.6%), and sterilization method 4 (0.8%). Concerning the knowledge of study participants towards the use of modern family planning methods, most of the study participants 507 (91.4%) knew at least one type of modern FP method.

### 3.4. Modern Contraceptive Use among Study Participants

The study finding showed that 243 (43.8%) of study participants had ever used modern contraceptive methods. One hundred and two (18.4%) women currently used modern contraceptive methods. Among the modern contraceptive methods, 50 (49%) women said they were using injectable methods, 45 (44.1%) said they used implants, 5 (4.9%) used pills, and 2 (2%) used IUCD. However, none of the participants reported the use of condoms and permanent contraceptive methods. Most of the study participants were using current modern contraceptive methods for spacing 81 (79.4%), for limiting birth 14 (13.4%), and for health benefits 7 (6.9%).

**Figure 2:**
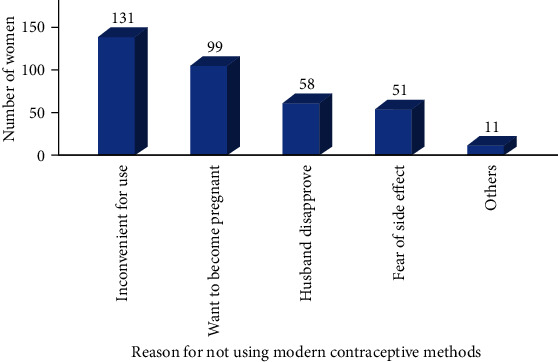
Reason for not using modern contraceptive methods among the currently married reproductive age group women in selected rural areas of Eastern Hararghe zone, Eastern Ethiopia, January 1-30, 2019 (*n* = 312).

When asked the reason for not using modern contraceptive methods, most of the study participants cited inconvenient for use 131 (41.9%) followed by want to become pregnant 99 (31.7%) (Figure 2).

### 3.5. Sexual and Reproductive Health Communication and Decision-Making on Modern FP Methods

Two hundred eighty-eight (51.9%) of the respondents said they believed that a couple's discussion is important in making a joint decision regarding modern contraceptive methods. Two hundred sixty-three (47.4%) of the interviewed women perceived that their husband supports the use of a modern contraceptive method. Three hundred twenty-nine (59.3%) of the women said that the male partner/husband should be involved in FP decision-making. 61.3% of interviewed women replied that they made decisions jointly with their husbands, while 30.4% of the interviewed women made decisions alone on modern contraceptive method use (Table 2).

### 3.6. Misconception about Modern FP Methods

Myths and misconceptions spread very easily in the community and discourage many potential or current users of family planning. Generally, 114 (20.5%) of the women perceived that using any type of modern contraceptive is a sin or against religion. The study also showed that 122 (22%) women responded that the use of contraceptive pills causes infertility. 29.2% of interviewed women feel that the injectable method causes infertility, and 25.2% feel that it causes menstrual disorders. Concerning implants, 226 (40.7%) perceived that they cause a menstrual disturbance and 78 (14.1%) of the women replied that IUCD causes infertility. Regarding condoms, 111 (20%) women responded that using condoms is a sign of promiscuity (Table 3).

### 3.7. Factors Associated with Modern Contraceptive Method Utilization

The findings from the multiple logistic regression analysis revealed that couple's discussion, the desire for additional child, male involvement in the decision of family planning, husband approval, ever use of the modern contraceptive method, and knowledge of modern contraceptive methods were independently associated factors of modern contraceptive utilization (Table 4).

## 4. Discussion

This study showed that modern FP utilization in the study area was very low despite universal knowledge of modern contraceptive methods by the community. The study revealed the overall modern FP utilization among the study participants was 18.4%. This finding is consistent with the study conducted in Bale zone, Southeast Ethiopia (20.8%) [12], but much higher than that of Afar Region, Eastern Ethiopia (8.5%) [13]. The variation might be due to the study population, as the first study was conducted among the pastoralist community.

The low use of modern FP in the current study is below that of the national survey (35%) [1]. The low coverage in the study area could be due to the influence of husbands, cultural taboos, and religious prohibition in an area where most (84.9%) of study participants are Muslims. Moreover, this study was conducted in a rural area with little public health and education infrastructure and high misperceptions towards modern contraception, compared to urban areas.

The study revealed that 91.4% of study participants knew at least one modern FP method. This finding is in line with a study finding from Bale zone (95.3%), Southeast Ethiopia [12], and Jimma zone (94%), Ethiopia [14]. This higher percent of information might be due to house-to-house FP information dissemination by health extension workers.

Our study finding is relatively higher than that of Dembia District (78.1%), Northwest Ethiopia [15], and Afar Region (62%), Eastern Ethiopia [13], but lower than studies from Uganda (98.1%) [16], Tanzania (98.8%) [17], and Butajira (99%) in Central Ethiopia [18] which reported almost universal knowledge of modern FP methods. The difference may be attributed to the wide variation in culture and socioeconomic characteristics of study participants.

Our study showed that women who know modern FP methods were about seventeen times (AOR = 16.958, CI: 4.768, 60.316) more likely to utilize modern FP methods than those women who do not know any type of modern contraceptive. This study finding is in line with the study finding from Awi zone, Amhara regional state, and the town of Debre Markos, Northwest Ethiopia [19, 20]. This can be justified by the fact that better knowledge of modern FP may result in better practice of modern FP methods, i.e., the women who know modern FP methods are more likely to use the method consistently and effectively than their counterparts.

In our study, the husband's view and approval of modern contraceptives showed an independent effect on modern contraceptive's method utilization. The odds of modern FP utilization among women who perceived that their husbands support the use of modern contraception were more than three times (AOR = 3.590, CI: 2.170, 5.936) higher than those women who do not perceive that their husbands support the use of modern contraceptives. The study finding is similar to findings from Ghana, Cameroon, and Bangladesh [21–23].

This study showed that women who discussed with their husbands about the issue of FP or SRH were about three times (AOR = 2.852, CI: 1.759, 4.623) more likely to use modern contraceptives than those women who did not discuss with their husbands. This finding is consistent with other studies conducted in Ghana, Ethiopia, and Tanzania [12, 15, 21, 24], and women whose partners were involved in FP decision-making were more than twice (AOR = 2.340, CI: 1.531, 3.576) more likely to use modern contraceptive methods than their counterparts. This finding is consistent with findings from Cameroon, Debre Markos, Northwest Ethiopia, and Zambia [20, 22, 25] and suggests that male involvement in decision-making improves spousal communication and decreases male opposition. This might be because women who discuss FP issues with their spouses may have their partner's approval on family planning; hence, they are more likely to use a modern method of contraception. This evidence is supported by a study conducted in the pastoralist community of Ethiopia that suggests that contraceptive use was influenced by both individual-/community-level characteristics [26]. This can be due to the fact that joint decisions for family planning are based on spousal communication implying that when couples communicate effectively, they are more likely to jointly agree on what type of contraceptive method to use, how many children to have, and the space between their offspring.

The current study reported that the odds of modern contraceptive utilization were more than two times (AOR = 2.295, CI: 1.528, 3.447) higher for women who do not desire an additional child than for those women who desire to have an additional child. This finding is similar to a study conducted in Zambia [27]. This is supported by responses from study participants, most of whom said they were using modern contraceptive methods for spacing and limiting birth.

The study also showed that women who had never used modern FP were only 0.018 times less likely to use modern FP methods, compared to those who had ever used modern FP methods (AOR = 0.018, CI: 0.005, 0.063), a similar finding to a study conducted in Uganda [28]. This can be due to the fact that ever users of modern FP methods were more familiar with the benefits of modern FP methods. Hence, they are more likely to use/continue the methods than their counterparts.

## 5. Limitations

Data were cross-sectional and originated from self-reported measures. In this case, it is difficult to validate claims made by respondents in the course of questionnaire administration. Moreover, there was a lack of contextual factors related to the health system, culture, religion, and societal norms of the study setting, which may affect modern FP use.

## 6. Conclusion

The present study contributes to our understanding of the determinants of modern FP utilization among women in rural Ethiopia. The overall prevalence of modern FP use among the study participants was low. Couple's discussion, the desire for additional child, male involvement in decision about family planning, husband approval, ever use of contraception, and knowledge of modern FP methods were found as significant factors that can increase modern FP method use.

The results of this survey suggest that the government should focus on positive action able to increase access to modern FP methods and it should create awareness of modern FP methods through mass media, community-based education, and proper counselling. The government should emphasize empowering women to make appropriate choices and address real barriers to women's use of modern FP methods.

## Figures and Tables

**Table 1:** Sociodemographic characteristics of currently married reproductive age group women in selected rural areas of Eastern Hararghe zone, Eastern Ethiopia, January 1-30, 2019 (*n* = 555).

Variables	Frequency (*N*)	Percentages (%)
Age		
15-24	138	24.9
25-34	304	54.8
35-49	113	20.4
Ethnicity		
Oromo	459	82.7
Amhara	51	9.2
Harari	25	4.5
^∗^Others	20	3.6
Religion		
Muslim	471	84.9
Orthodox	51	9.2
Protestant	21	3.8
^∗∗^Others	12	2.1
Education		
No formal education	307	55.3
Primary school	195	35.1
Secondary school	34	6.1
10+	19	3.4
Average monthly income		
<1000 ETB	242	43.6
1000-1999 ETB	207	37.3
2000-2999 ETB	83	15.0
3000-3999 ETB	23	4.1

^∗^Others: Gurage, Tigre, and Somali; ^∗∗^others: Catholic, Adventists, and Pagans; ETB: Ethiopian birr.

**Table 2:** Sexual and reproductive health (SRH) communication and decision-making of modern FP methods among currently married reproductive age group women in selected rural areas of Eastern Hararghe zone, Eastern Ethiopia, January 1-30, 2019 (*n* = 555).

Variables	Frequency (*N*)	Percentages (%)
Discussion on SRH/FP		
Yes	288	51.9
No	267	48.1
Do you think that a couple's discussion on SRH/FP is important?		
Yes, very important	308	55.5
Somewhat important	94	16.9
Little important	29	5.2
Not important at all	124	22.3
Do you think that the partner should be involved in the FP decision?		
Yes	329	59.3
No	226	40.7
What is your partner's idea towards modern contraceptive method use?		
Supportive	263	47.4
Neutral	60	10.8
Not supportive	232	41.8
Who decides on contraceptive use?		
Joint decision	141	61.3
Mainly my decision	70	30.4
Mainly husband decision	14	6.1
^∗^Others	5	2.2

^∗^Others: relatives, friends, and health care providers.

**Table 3:** Misconceptions towards modern FP methods among currently married reproductive age group women in selected rural areas of Eastern Hararghe zone, Eastern Ethiopia, January 1-30, 2019 (*n* = 555).

Contraceptive types	Misconceptions	Frequency (*N*)	Percentage (%)
Pills	Causes infertility	122	22
	Sinful/against religion	114	20.5
	Menstrual disorder	93	16.8
	Causes headache	75	13.5
	Affects baby/abortion	25	4.6
	Premature menopause	15	2.7

Injectable	Causes infertility	162	29.2
	Menstrual disorder	140	25.2
	Sinful/against religion	114	20.5
	Causes headache	75	13.5
	Premature menopause	50	9
	Causes abortion	18	3.2
	Causes cancer	16	2.9

Implants	Menstrual disorder	226	40.7
	Causes infertility	162	29.2
	Sinful/against religion	114	20.5
	Premature menopause	50	9
	Causes abortion	18	3.2
	Causes cancer	16	2.9

IUCD	Sinful/against religion	114	20.5
	Causes infertility	78	14.1
	Migrates to the head	29	5.2
	Damage the uterus	19	3.4
	Causes cancer	15	2.7
	Causes abortion	7	1.3

Condom	Sign of promiscuity	111	20
	Discomfort during sex/painful	26	4.7
	For unmarried only	19	3.4
	Decrease sexual performance, sexual pleasure, sexual desire/libido	15	2.7

**Table 4:** Multivariate analysis of factors associated with modern FP method utilization among currently married reproductive age group women in selected rural districts of Eastern Hararghe zone, Eastern Ethiopia, January 1-30, 2019 (*n* = 555).

Variables	Use of modern FP methods		Odds ratio (95% confidence interval)	
	Yes	No	COR (95% CI)	AOR (95% CI)
Age				
18-25	46	92	1	1
26-40	197	220	1.791 (1.197, 2.679)	0.691 (0.402, 1.188)
Average monthly income				
≤2000	40	32	1.724 (1.047, 2.839)	1.309 (0.724, 2.365)
>2000	203	280	1	1
Number of pregnancies				
0-4	114	205	1	1
5 and above	129	107	2.168 (1.537, 3.057)	0.666 (0.368, 1.204)
Ever use of modern FP method				
Yes	99	144	1	1
No	3	309	0.014 (0.004, 0.045)	0.018 (0.005, 0.063)^∗^
Desire for additional child				
Yes	159	174	1	1
No	84	138	2.387 (1.688, 3.374)	2.295 (1.528, 3.447)^∗^
Couple's discussion				
Yes	177	111	4.856 (3.369, 6.999)	2.852 (1.759, 4.623)^∗^
No	201	66	1	1
Partner involvement in decision-making				
Yes	131	198	1.485 (1.055, 2.090)	2.340 (1.531, 3.576)^∗^
No	112	114	1	1
Partner attitude				
Supportive	172	91	6.230 (4.191, 9.263)	3.590 (2.170, 5.936)^∗^
Neutral	17	43	1.303 (0.688, 2.468)	0.922 (0.454, 1.874)
Not supportive	54	178	1	1
Knowledge of modern FP methods				
Knowledgeable				
Yes	240	267	13.483 (4.136, 43.951)	16.958 (4.768, 60.316)^∗^
No	3	45	1	1

^∗^Significant at *P* < 0.05 in multivariate analysis.

## Data Availability

The data used to support the findings of this study are available from the corresponding author after formal communication by email, if the request is for an acceptable reason.
